# Supplementary material for: A Comprehensive Assessment of Ultraviolet-Radiation-Induced Mutations in Flammulina filiformis Using Whole-Genome Resequencing
Source: J Fungi (Basel). 2024 Mar 20;10(3):228. doi: 10.3390/jof10030228 (PMC10971301; doi:10.3390/jof10030228)
Supplement: Supplementary file 1 [file jof-10-00228-s001.zip › Supplementary Material S8/KEGG annotation/out/64381550635650.os/KO/out_map/map04622.html]

KEGG PATHWAY: RIG-I-like receptor signaling pathway - Reference pathway


|  |  |
| --- | --- |
| **RIG-I-like receptor signaling pathway - Reference pathway** |  |

[
Pathway menu
| Organism menu
| Pathway entry
| Show description
| User data mapping
]

Specific families of pattern recognition receptors are responsible for detecting viral pathogens and generating innate immune responses. Non-self RNA appearing in a cell as a result of intracellular viral replication is recognized by a family of cytosolic RNA helicases termed RIG-I-like receptors (RLRs). The RLR proteins include RIG-I, MDA5, and LGP2 and are expressed in both immune and nonimmune cells. Upon recognition of viral nucleic acids, RLRs recruit specific intracellular adaptor proteins to initiate signaling pathways that lead to the synthesis of type I interferon and other inflammatory cytokines, which are important for eliminating viruses.

|  |  |
| --- | --- |
| Reference pathway | 100% |
